# High mobility group box-1 protein in patients with suspected community-acquired infections and sepsis: a prospective study

**DOI:** 10.1186/cc5715

**Published:** 2007-03-08

**Authors:** Shahin Gaïni, Svend Stenvang Pedersen, Ole Græsbøll Koldkjær, Court Pedersen, Holger Jon Møller

**Affiliations:** 1 Department of Infectious Diseases, Odense University Hospital, Søndre Boulevard 29, DK-5000 Odense C, Denmark; 2 Department of Clinical Biochemistry, Sønderborg Hospital, Sydvang 1, DK-6400 Sønderborg, Denmark; 3 Department of Clinical Biochemistry, Aarhus University Hospital, Nørrebrogade 44, DK-8000 Aarhus C, Denmark

## Abstract

**Introduction:**

Sepsis is a serious condition with a significant morbidity and mortality. New insight into the immunopathogenesis of sepsis could promote the development of new strategies for diagnosis and therapy. High mobility group box-1 protein (HMGB1) has been known for many years as a nuclear chromosomal protein. Its role as a pro-inflammatory cytokine in sepsis and rheumatoid arthritis has been described recently. The aim of our study was to evaluate HMGB1 as a molecular marker in patients with community-acquired infections.

**Methods:**

Patients suspected of having infections/sepsis and admitted to a department of internal medicine were included in the study in a prospective manner. Demographic data, comorbidity, routine biochemistry, microbiological data, infection focus, severity score, and mortality on day 28 were recorded. Plasma and serum were sampled at the time of admission. HMGB1 levels were measured with a commercially available enzyme-linked immunosorbent assay (ELISA). Procalcitonin levels were measured with a TRACE (time-resolved amplified cryptate emission) assay. Lipopolysaccharide-binding protein and interleukin-6 were measured with a chemiluminiscent immunometric assay. Soluble haemoglobin scavenger receptor (sCD163) levels were measured with an in-house ELISA.

**Results:**

One hundred and ninety-four patients were included in the study. Levels of HMGB1 are presented as medians and interquartile ranges: healthy controls (0.77 ng/ml, 0.6 to 1.46), non-infected patients (1.54 ng/ml, 0.79 to 2.88), infected patients without systemic inflammatory response syndrome (2.41 ng/ml, 0.63 to 3.44), patients with sepsis (2.24 ng/ml, 1.30 to 3.75), and patients with severe sepsis (2.18 ng/ml, 0.91 to 3.85). In a receiver operator characteristic curve analysis discriminating between non-infected patients and all infected patients, the area under the curve for HMGB1 was 0.59 (*P *< 0.0001). HMGB1 correlated only weakly to levels of white blood cell count, neutrophils, C-reactive protein, interleukin-6, procalcitonin, and lipopolysaccharide-binding protein (*P *< 0.001). HMGB1 did not correlate to sCD163.

**Conclusion:**

In a cohort of patients with suspected community-acquired infections and sepsis, HMGB1 levels were statistically significantly higher in patients compared to the healthy controls. There was no statistically significant difference between the infected and the non-infected patients. Levels of HMGB1 correlated only very weakly to other pro-inflammatory markers and did not correlate to the anti-inflammatory marker sCD163.

## Introduction

Sepsis is a serious condition with a significant morbidity and mortality [1]. Clinicians treating patients with infections and sepsis are in need of better diagnostic, prognostic, and immunological molecular markers. Better markers for the presence of infection and the degree of inflammation would enable the clinician to start relevant therapy as early as possible. Increased insight into the immunopathogenesis of sepsis would offer the potential to generate new treatment options. Sepsis is characterised by an activation of the innate immune system when the immune system is challenged by an invading pathogenic microorganism [2]. This results in the production of pro-inflammatory and anti-inflammatory cytokines [2].

A lot of attention has been given to several pro-inflammatory cytokines involved in sepsis. Cytokines like interleukin (IL)-1, IL-6, and tumour necrosis factor-alpha have been studied in animal models and in clinical sepsis cohorts [3-9]. These cytokines have an important role in initiating the systemic inflammatory response syndrome (SIRS) in the early phases of sepsis. In a laboratory model with cultured macrophages stimulated with endotoxin, high mobility group box-1 protein (HMGB1) was identified as a potential 'late-onset' pro-inflammatory cytokine [10]. It was also observed that mice had increased levels of HMGB1 8 to 32 hours after exposure to endotoxin [10]. Treatment with antibodies against HMGB1 reduced mortality in endotoxin-exposed mice, and administration of HMGB1 increased mortality [10].

HMGB1 levels have been studied in critically ill patients. However, the studies were characterised by few patients in heterogenic patient populations. Measuring HMGB1 has been quite challenging because no enzyme-linked immunosorbent assay (ELISA) was available until recently. Earlier studies used blotting methods for measuring HMGB1. We have previously shown that C-reactive protein (CRP) and IL-6 were better markers for infection than soluble haemoglobin scavenger receptor (sCD163) in a population of patients prospectively admitted to a department of internal medicine [11]. We have also shown that CRP, lipopolysaccharide-binding protein (LBP), and IL-6 were better diagnostic markers for infection and sepsis than procalcitonin (PCT) in the same cohort of patients [12]. The purpose of the present study was to describe levels of HMGB1 in a non-critically ill population of patients suspected of having sepsis. To perform the study, we used the patient cohort used in two previous studies [11,12].

## Materials and methods

### Patients

Patients admitted to the department of internal medicine were consecutively included in our study in a five month period in 2003. Odense University Hospital (Odense, Denmark) has 1,200 beds and serves a local population of 185,000 inhabitants. Inclusion criteria were suspected diagnosis of infection as judged by the referring physician and blood cultures drawn at the time of admission. Exclusion criteria were age below 18 years, earlier participation in the study, or prior hospitalisation within seven days before admission. Plasma and serum were sampled immediately after admission. The samples were processed and frozen at -80°C within 1.5 hours after sampling. Sampling was performed before the administration of any antibiotics was started at the hospital. Informed consent was obtained from all patients or from their close relatives. The project was approved by the Ethics Committee of Vejle and Fyns counties.

Baseline characteristics, demographic characteristics, routine biochemical data, SIRS criteria, and severity score were obtained at the time of inclusion. Comorbidity was assessed with the Charlson Index [13]. Severity was assessed with the Sepsis-related Organ Failure Assessment (SOFA) score [14]. Patients were assessed with the SIRS criteria at the time of admission [15]. Severe sepsis was defined as the presence of sepsis combined with one or more of the following: Glasgow Coma Scale of less than or equal to 14, PaO_2 _(partial pressure of oxygen, arterial) of less than or equal to 9.75 kPa, oxygen saturation of less than or equal to 92%, PaO_2_/FiO_2 _(fraction of inspired oxygen) of less than or equal to 250, systolic blood pressure of less than or equal to 90 mm Hg, systolic blood pressure decrease of more than or equal to 40 mm Hg from baseline, pH of less than or equal to 7.3, lactate of more than or equal to 2.5 mmol/l, creatinine of more than or equal to 177 μmol/l, 100% increase of creatinine in patients with known kidney disease, oliguria of less than or equal to 30 ml/hour in more than three hours or of less than or equal to 0.7 litres per 24 hours, prothrombin time of less than or equal to 0.6 (reference: 0.70–1.30), platelets of less than or equal to 100 × 10^9^/l, bilirubin of more than or equal to 43 μmol/l, and paralytic ileus. Septic shock was defined as hypotension persisting despite adequate fluid resuscitation for at least one hour. The criteria were not valid if any comorbidity could more relevantly explain them. The presence of infection was defined by at least one of the following: identification of a pathogenic microorganism by cultures or polymerase chain reaction, pneumonia verified by chest x-ray, infection documented by other imaging technique, serologically documented infection, and obvious clinical infection (for instance, erysipelas and wound infection). The physician classifying the infection status of the included patients was blinded to all biochemical laboratory results. The patients were divided into the following groups for analyses: non-infected patients, infected patients without SIRS, sepsis patients, and patients with severe sepsis/septic shock. Patients who could not be classified were excluded from the analyses.

### Laboratory assays

HMGB1 was measured with a commercially available ELISA (HMGB1 ELISA kit; Shino-Test Corporation, Tokyo, Japan). The measuring range was 0.6 to 93.8 ng/ml. The range could be broadened by dilution of high samples. The coefficients of variation were 5% for samples above 10 ng/ml and 10% for samples from 2 to 5 ng/ml. Recovery of HMGB1 in this ELISA was 92% to 111% [16]. PCT was measured with a TRACE (time-resolved amplified cryptate emission) technology assay (Kryptor PCT^®^; B·R·A·H·M·S Aktiengesellschaft, Hennigsdorf, Germany). The functional assay sensitivity was 0.06 ng/ml. LBP and IL-6 were measured with a chemiluminiscent immunometric assay (Immulite-1000^®^; Diagnostic Products Corporation, Los Angeles, CA, USA). The detection limit of LBP was 0.2 μg/ml. The detection limit of IL-6 was 2 pg/ml. sCD163 was measured with an in-house ELISA as described previously [17]. CRP was measured with an immunoturbidimetric principle (Modular P^®^; Hitachi, Ltd., Tokyo, Japan). White blood cells (WBCs) and neutrophils were counted on a Sysmex SE 9000^® ^(TOA Corporation, Kobe, Japan).

### Statistical analysis

Data are presented as medians and interquartile ranges (IQRs) and as means ± standard deviations. Significance testing was performed using the Kruskal-Wallis test and the Wilcoxon two-sample test. A two-tailed *P *value of less than 0.05 was considered statistically significant. Receiver operator characteristic (ROC) curves and the area under the curve (AUC) were calculated for all the examined inflammatory markers. Ninety-five percent confidence intervals (CIs) were reported for the AUC. The method described by DeLong and colleagues [18] was used as the significance test for ROC curve and AUC comparison. The Spearman rank correlation test was used to determine correlations. At the time of the study, our clinical biochemistry department did not report CRP levels below 10 mg/l. CRP levels below 10 mg/l were therefore assigned a value of 10 mg/l for calculations. The detection limit of the HMGB1 ELISA was 0.6 ng/ml. HMGB1 levels below 0.6 ng/ml were therefore assigned a value of 0.6 ng/ml for calculations. The detection limit of the IL-6 assay was 2 pg/ml. IL-6 measurements below 2 pg/ml were therefore assigned a value of 2 pg/ml for calculations. All statistical calculations were performed with the STATA 8^® ^statistical software package (StataCorp LP, College Station, TX, USA).

## Results

### Patient characteristics

One hundred and ninety-four patients were included in the study. The patients were divided according to our plan for analyses into the following groups: non-infected patients (*n *= 67), infected patients without SIRS (*n *= 32), patients with sepsis (*n *= 47), and patients with severe sepsis (*n *= 27). Twenty-one patients could not be classified and were excluded from analyses. Only one patient had septic shock. This patient was included in the severe sepsis group. The diagnoses of the non-infected patients were respiratory disease (*n *= 22), cardiovascular disease (*n *= 10), rheumatologic disease (*n *= 8), central nervous system disease (*n *= 5), and various other diseases (*n *= 22). Sixteen patients in the non-infected group were treated with immunosuppressive drugs at the time of admission (15 with prednisolone and 1 with methotrexate). Fifteen of the infected patients (with or without SIRS) were treated with prednisolone at the time of admission. All but one of these patients continued on their immunosuppressive treatment during their hospital stay. The mortality rate among all the infected patients was 3.8%. Thirty-two healthy hospital workers served as a healthy control group for HMGB1 analyses. Baseline characteristics and mortality at day 28 are presented in Table 1. The microbiology and infectious focus are presented in Table 2.

**Table 1:** Baseline characteristics and outcome of the patients

Variable	Non-infected patients (*n *= 67)	Infected patients without SIRS (*n *= 32)	Patients with sepsis (*n *= 47)	Patients with severe sepsis (*n *= 27)
Gender, number (percentage)				
Male	23 (34.3)	18 (56.3)	20 (42.6)	18 (66.7)
Female	44 (65.7)	14 (43.7)	27 (57.4)	9 (33.3)
Age in years, mean ± SD	67.3 ± 17.1	60.8 ± 16.6	60.4 ± 19.9	66.4 ± 17.8
Length of hospitalisation in days, mean ± SD	8.5 ± 6.9	10.3 ± 11.5	7.8 ± 6.7	10.8 ± 10.5
Mortality on day 28, number (percentage)	6 (8.9)	0	0	4 (14.8)
Severity of disease, mean ± SD				
SOFA score	1.4 ± 1.1	1.6 ± 1.5	1.6 ± 1.2	3.0 ± 1.9
Comorbidity, mean ± SD				
Charlson Index	1.6 ± 1.3	1.3 ± 1.3	1.1 ± 1.3	1.2 ± 1.3
Laboratory findings, mean ± SD				
Haemoglobin, mmol/l	8.1 ± 1.1	8.2 ± 1.2	8.2 ± 1.2	8.2 ± 1.1
Platelet count, 10^9^/l	288.5 ± 108.2	324.5 ± 210.6	254.4 ± 107.3	268.0 ± 184.4
Bilirubin, μmol/l	9.3 ± 6.9	21.9 ± 36.6	10.6 ± 6.8	13.6 ± 5.5
Prothrombin time (reference: 0.70–1.30)	1.0 ± 0.3	0.9 ± 0.4	0.9 ± 0.3	0.9 ± 0.3
Creatinine, μmol/l	96.7 ± 27.3	100.6 ± 31.2	100.4 ± 31.7	140.3 ± 79.5

SD, standard deviation; SIRS, systemic inflammatory response syndrome; SOFA, Sepsis-related Organ Failure Assessment.

**Table 2:** Microbiological and infection characteristics of the patients

Variable	Infected patients without SIRS (*n *= 32)	Patients with sepsis (*n *= 47)	Patients with severe sepsis (*n *= 27)
Assessment of infection, number			
Gram-positive bacteria	6	12	10
Gram-negative bacteria	7	10	7
Other bacteria	0	2^a^	0
Bacteraemia	1	4	7
Virus	3^b^	4^c^	1^d^
CXR verified pneumonia^e^	9	13	7
Radiological evidence^f^	0	1	0
Obvious clinical infection^g^	7	5	2
Focus of infection, number			
Upper respiratory tract infection	1	0	1
Lower respiratory tract infection	12	25	15
Endocarditis	1	0	1
Gastroenteritis	5	1	0
Pyelonephritis	2	2	1
Cystitis	0	3	3
Skin/Soft tissue infection	1	4	3
Bone/Joints	2	1	0
Other	8	11	3

^a^*Mycoplasma pneumoniae *(*n *= 2); ^b^Epstein-Barr virus (*n *= 1), influenza A virus (*n *= 2);

^c^Epstein-Barr virus (*n *= 2), influenza A virus (*n *= 2); ^d^Puumala virus (*n *= 1); ^e^CXR verified pneumonia with no identified pathogen; ^f^infection documented by imaging techniques (other than CXR) with no identified pathogen; ^g^clinical infection (for instance, erysipelas, wound infection). CXR, chest x-ray; SIRS, systemic inflammatory response syndrome.

### Levels of high mobility group box-1 protein

**Figure 1:**
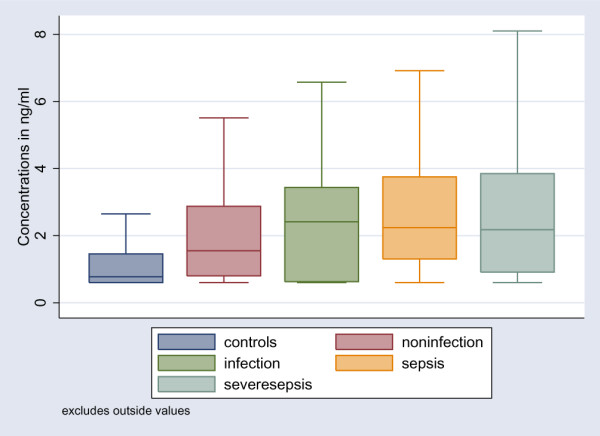
Boxplot of HMGB1 levels in healthy controls, non-infected patients (*P *< 0.001 compared to healthy controls), infected patients without systemic inflammatory response syndrome (SIRS) (*P *= 0.32 compared to non-infected patients), patients with sepsis (*P *= 0.48 compared to infected patients without SIRS), and patients with severe sepsis (*P *= 0.37 compared to patients with sepsis). HMGB1, high mobility group box-1 protein.

**Figure 2:**
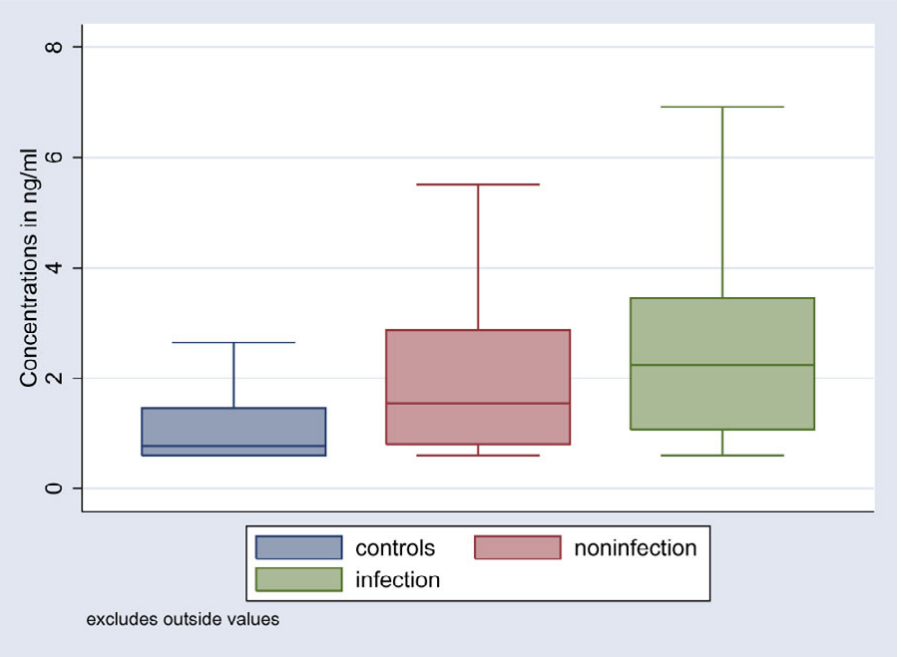
Boxplot of HMGB1 levels in healthy controls, non-infected patients (*P *< 0.001 compared to healthy controls), and all infected patients (*P *= 0.054 compared to non-infected patients). HMGB1, high mobility group box-1 protein.

**Figure 3:**
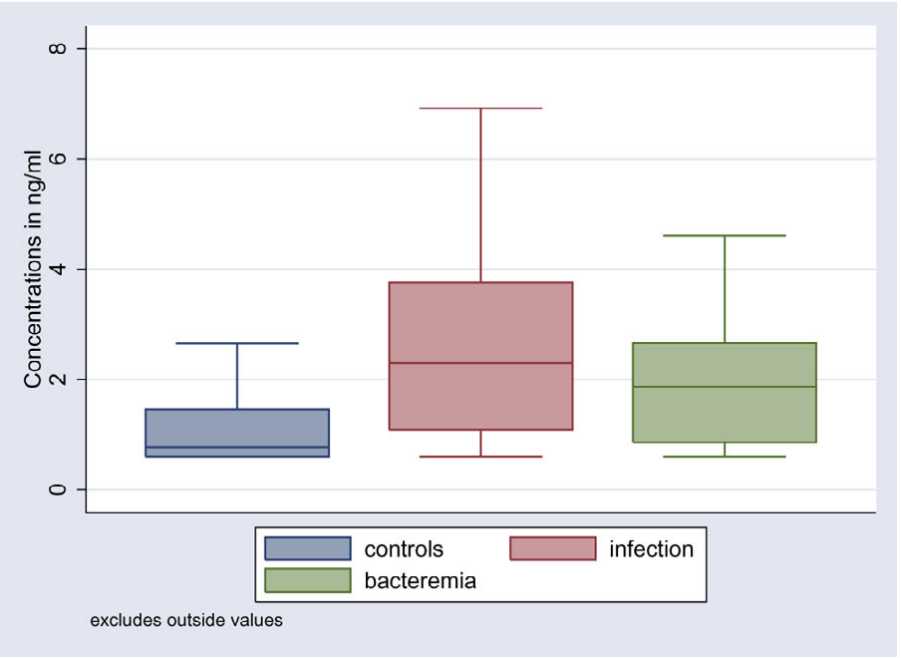
Boxplot of HMGB1 levels in healthy controls, infected patients without bacteraemia (*P *< 0.0001 compared to healthy controls), and patients with bacteraemia (*P *< 0.05 compared to healthy controls; *P *= 0.38 compared to infected patients without bacteraemia). HMGB1, high mobility group box-1 protein.

The levels of HMGB1 were statistically significantly higher among patients compared to the healthy control group (*P *< 0.001) (Table 3). However, there were no statistically significant differences in HMGB1 levels between the following groups: non-infected patients, infected patients without SIRS, patients with sepsis, and patients with severe sepsis (Table 3; Figure 1). When all infected patients (infection without SIRS, sepsis, and severe sepsis) as a group were compared with the non-infected patients, the difference was marginally significant (*P *= 0.054) (Figure 2). Levels of HMGB1 were significantly higher in infected patients (infection without SIRS, sepsis, and severe sepsis) without bacteraemia (*n *= 94) and in patients with bacteraemia (*n *= 12) compared to healthy controls (Figure 3). Levels of HMGB1 were higher among non-infected patients treated with immunosuppressive drugs (median 2.8 ng/ml) compared to non-infected patients not treated with immunosuppressive drugs (median 1.5 ng/ml) (*P *< 0.05). There were no statistically significant differences in HMGB1 levels between infected patients treated with immunosuppressive drugs and infected patients not treated with immunosuppressive drugs. Levels of CRP, PCT, LBP, and IL-6 were statistically significantly higher among all infected patients compared to the non-infected patients (Table 3). Levels of sCD163 were statistically significantly higher only in the group with severe sepsis compared to the non-infected patients (Table 3). Levels of WBC count and neutrophils were statistically significantly higher among patients with sepsis and severe sepsis compared to the non-infected patients (Table 3).

**Table 3:** Levels of HMGB1, PCT, LBP, CRP, IL-6, WBC, and neutrophils in different groups

Variable	Healthy controls (*n *= 32)	Non-infected patients (*n *= 67)	Infected patients without SIRS (*n *= 32)	Patients with sepsis (*n *= 47)	Patients with severe sepsis (*n *= 27)	*P *value^a^
HMGB1 (ng/ml)						< 0.001
Median	0.77	1.54	2.41	2.24	2.18	
IQR	0.6–1.46	0.79–2.88	0.63–3.44	1.30–3.75	0.91–3.85	
PCT (ng/ml)						< 0.001
Median		0.09	0.16	0.2	1.9	
IQR		0.05–0.13	0.07–0.34	0.08–0.65	0.22–14.6	
LBP (μg/ml)						< 0.001
Median		16.3	27.4	33.5	40.4	
IQR		12.05–25.3	18.3–41.2	25.0–43.2	18.0–63.6	
CRP (mg/l)						< 0.001
Median		18	122.0	120.0	217.0	
IQR		10–42	54.0–215.0	41.0–190.0	78.0–414.0	
IL-6 (pg/ml)						< 0.001
Median		9.8	20.6	72.6	199.3	
IQR		2.85–21.65	9.8–99.4	25.9–274.5	67.5–2,833.0	
WBC count (10^9^/l)						< 0.001
Median		8.2	9.5	13.0	12.2	
IQR		6.7–10.3	7.7–11.9	9.2–17.1	7.0–17.5	
Neutrophils (10^9^/l)						< 0.001
Median		6.19	7.1	10.1	10.3	
IQR		4.73–7.9	5.1–9.7	7.1–14.8	5.5–15.4	
sCD163 (mg/l)						0.06
Median		2.99	3.62	3.17	3.63	
IQR		2.21–4.05	2.44–4.54	2.27–4.64	2.67–5.72	

^a^Kruskal-Wallis test. CRP, C-reactive protein; HMGB1, high mobility group box-1 protein; IL-6, interleukin-6; IQR, interquartile range; LBP, lipopolysaccharide-binding protein; PCT, procalcitonin; sCD163; soluble haemoglobin scavenger receptor; SIRS, systemic inflammatory response syndrome; WBC, white blood cell.

### Receiver operating characteristic curve

**Figure 4:**
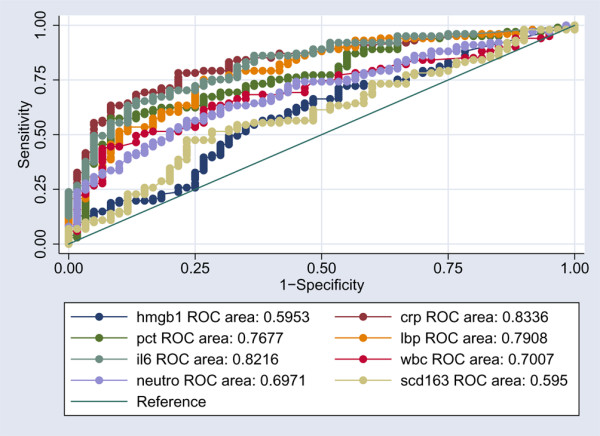
Receiver operating characteristic (ROC) curves comparing the discriminating capabilities of high mobility group box-1 protein (HMGB1), C-reactive protein (CRP), procalcitonin (PCT), lipopolysaccharide-binding protein (LBP), interleukin-6 (IL-6), white blood cell (WBC), neutrophils (neutro), and soluble haemoglobin scavenger receptor (sCD163) between non-infected patients and all infected patients (*P *< 0.0001).

In an ROC curve analysis to distinguish between the non-infected patients and all infected patients (infection without SIRS, sepsis, and severe sepsis), the markers performed with the following AUCs: HMGB1 0.59 (95% CI 0.5 to 0.68), CRP 0.83 (95% CI 0.77 to 0.89), PCT 0.76 (95% CI 0.69 to 0.84), LBP 0.79 (95% CI 0.72 to 0.86), IL-6 0.82 (95% CI 0.76 to 0.88), sCD163 0.59 (95% CI 0.5 to 0.68), WBC 0.70 (95% CI 0.62 to 0.78), and neutrophils 0.69 (95% CI 0.62 to 0.77). HMGB1 and sCD163 thus performed poorest in this comparative ROC curve analysis (Figure 4).

### Correlations

Weak correlations were found between HMGB1 and CRP (Spearman rank correlation coefficient *r *= 0.27, *P *< 0.001), HMGB1 and PCT (Spearman rank correlation coefficient *r *= 0.17, *P *< 0.05), HMGB1 and LBP (Spearman rank correlation coefficient *r *= 0.26, *P *< 0.001), and HMGB1 and IL-6 (Spearman rank correlation coefficient *r *= 0.21, *P *< 0.01). Correlations of moderate strength were found between HMGB1 and WBC (Spearman rank correlation coefficient *r *= 0.36, *P *< 0.0001) and HMGB1 and neutrophils (Spearman rank correlation coefficient *r *= 0.42, *P *< 0.0001). No correlation was found between HMGB1 and sCD163.

## Discussion

The patients included in our study were representative of patients admitted to a department of internal medicine with the diagnosis of suspected infection. They were elderly patients with a considerable burden of comorbidity. Compared to patients in previous clinical studies focusing on markers of infection and sepsis, our patients were not as ill. This is shown by the relatively low mortality rate, low SOFA scores, and the fact that only one patient had septic shock. Our cohort was therefore dominated by the milder end of the sepsis spectrum. Most previous studies investigating different immunological aspects of sepsis have focused on patients admitted to intensive care units and thus on the more severely ill. We believe that focusing on patients in the milder end of the sepsis spectrum is a strength. In the early phase of infectious/sepsis disease, it is critical to have good diagnostic markers to identify patients in need of effective antibiotic therapy and other supportive care. It is also important to have good prognostic and immunological markers in these patients. If clinicians want to use clinical research results on their patients, the validation of (for instance) sepsis markers will ideally have been performed on a patient sample representative of the clinical reality that faces the clinician. Our study cohort was well characterised, and the sampling and processing of plasma/serum were optimised. We avoided work-up bias in the classification of the infectious status of the patients by blinding the clinicians and laboratory technicians. Drawbacks of this study (as of most other clinical sepsis studies) were heterogeneity among included patients, a heavy burden of comorbidity, and different length of disease prior to hospital admission. Another drawback was the risk of imperfect gold-standard bias in classifying the patients.

HMGB1 is a 215-amino acid protein that has been shown to be highly conserved among different species [19]. It has been known for approximately 30 years as a nuclear chromosomal protein [19,20]. In recent years, there has been a focus on a new role for HMGB1. It has been suggested that HMGB1 has a role as a pro-inflammatory cytokine [21], and HMGB1 has been shown to have many organ-specific biological functions, including inducing fever, anorexia, weight loss, and cytokine production in the brain; inducing acute lung injury and production of pro-inflammatory cytokines/mediators in the lungs; promoting translocation in the gut; inducing arthritis and joint inflammation; affecting heart rhythm; and having bactericidal effects [22].

To our knowledge, only three clinical studies with data on HMGB1 levels in infections and sepsis have been published [10,23,24]. In one small study with 8 healthy people and 25 patients with sepsis, the highest levels of HMGB1 (median 84 ng/ml) were observed in sepsis patients with a fatal outcome [10]. Surviving patients had a median level of HMGB1 of 25 ng/ml, and healthy controls had undetectable levels of HMGB1 [10]. In that study, HMGB1 levels were measured with an immunoblotting method [10]. In a prospective observational study, HMGB1 and several other cytokines were measured over several days in patients who had different degrees of sepsis and who were admitted to intensive care units [23]. In that study, HMGB1 was measured with two different immunoblotting methods [23]. Levels of HMGB1 remained elevated in this cohort of critically ill patients up to one week after inclusion [23]. Levels of HMGB1, in general, remained elevated for a longer time compared to other cytokines measured in the same cohort [23]. In a recent study, HMGB1 levels were measured with an ELISA in several groups of patients, some of them infected [24]. Mean levels of HMGB1 were as follows: undetectable in healthy controls, 4.54 ng/ml in infected patients, 2.15 ng/ml in patients with malignant disease, 6.47 ng/ml in trauma patients, and 14.05 ng/ml in patients with disseminated intravascular coagulation [24]. High levels were observed in patients with organ failure (mean 8.29 ng/ml) and in fatal cases (mean 16.58 ng/ml) [24]. It is difficult to compare the abovementioned studies; two of them used immunoblotting methods for measuring HMGB1 [10,23] and the other used an ELISA [24]. Our study data suggest that HMGB1 levels are much lower in patients in the milder end of the sepsis spectrum. It is possible that the low levels of HMGB1 in our study could be explained either by the fact that the patients were less ill or by the laboratory method we used to measure HMGB1. The presence of interfering inhibiting factors/autoantibodies to HMGB1 in human serum could affect the results of HMGB1 measurements with ELISA techniques [25]. It is still unknown whether the currently used assays detect biologically active HMGB1. This is an important issue in studies focusing on HMGB1 levels and disease activity. Another explanation could be that we sampled our patients early in their disease course, and this could explain why levels of a 'late-onset' cytokine would be low early after admission. As mentioned earlier, a drawback of the study design was the lack of data on the length of illness before admission. Our data showed no statistically significant difference between the non-infected patients and the infected patients. Our ROC curve analysis confirmed the abovementioned observation showing that HMGB1, in common with sCD163, performed poorly in ROC curve analysis (with an AUC of only 0.59). The trend of lower HMGB1 levels which we observed in the most severely ill patients (severe sepsis and bacteraemia) was observed earlier in one of the abovementioned studies [23]. Given the increasing focus on immune paresis as a possible mechanism in severe sepsis and septic shock, this is an interesting observation [26]. If HMGB1 is considered a strong pro-inflammatory cytokine involved in the pro-inflammatory phase of SIRS/sepsis, it could be hypothesised that lower levels could be observed when the patient with severe sepsis/septic shock moves from a pro-inflammatory state to a state with immune paresis. In our study, there was no correlation between an anti-inflammatory marker of sepsis (sCD163) and HMGB1.

## Conclusion

This is the first study focusing on HMGB1 levels in a cohort of patients suspected of having community-acquired infections and sepsis and admitted to a department of internal medicine. This cohort was dominated by patients with infections without SIRS, patients with sepsis, and patients with severe sepsis. These sepsis patients were in the mild end of the sepsis spectrum and had low SOFA scores and a low mortality rate. Sixty-seven of the patients were classified as non-infected patients and served as our main control group. Levels of HMGB1 were significantly higher in patients compared to healthy controls. There was no significant difference in levels between the non-infected patients and the infected patients (infection without SIRS, sepsis, and severe sepsis) (*P *= 0.054). HMGB1 levels correlated only very weakly to other pro-inflammatory markers (CRP, IL-6, PCT, LBP, WBC, and neutrophils). HMGB1 did not correlate to the anti-inflammatory marker sCD163. Our data do not suggest that HMGB1 has a role in differentiating between infected and non-infected patients admitted to a department of internal medicine. Further studies are needed to elucidate the role of HMGB1 in the immunopathogenesis of sepsis. Studies focusing on the kinetics of HMGB1 and consecutive measurements of HMGB1 should also be encouraged.

## Key messages

• The role of HMGB1 as a nuclear chromosomal protein has been known for many years.

• In recent years, the role of HMGB1 as an inflammatory cytokine has been explored.

• In a cohort of patients suspected of having community-acquired infections and sepsis, levels of HMGB1 were statistically significantly higher in patients compared to the healthy controls.

## Abbreviations

AUC = area under the curve; CI = confidence interval; CRP = C-reactive protein; ELISA = enzyme-linked immunosorbent assay; HMGB1 = high mobility group box-1 protein; IL = interleukin; IQR = interquartile range; LBP = lipopolysaccharide-binding protein; PaO_2 _= partial pressure of oxygen, arterial; PCT = procalcitonin; ROC = receiver operating characteristic; sCD163 = soluble haemoglobin scavenger receptor; SIRS = systemic inflammatory response syndrome; SOFA = Sepsis-related Organ Failure Assessment; WBC = white blood cell.

## Competing interests

The authors declare that they have no competing interests.

## Authors' contributions

SG planned the study, wrote the protocol, collected and analysed data, and wrote the report. OGK was responsible for PCT, IL-6, and LBP analyses. HJM developed the sCD163 in-house ELISA and was responsible for HMGB1 and sCD163 analyses. SSP and CP were involved in planning the study, revising the manuscript, and practical clinical aspects. All authors read and approved the final manuscript.
